# Work–Life Balance and Academic Productivity Among College of Medicine Faculty During the Evolution of the COVID-19 Pandemic: The New Normal

**DOI:** 10.1089/whr.2023.0007

**Published:** 2023-07-18

**Authors:** Heather M. Weinreich, Pavitra Kotini-Shah, Bernice Man, Ruth Pobee, Laura E. Hirshfield, Barbara J. Risman, Irina A. Buhimschi

**Affiliations:** ^1^ Department of Otolaryngology—Head and Neck Surgery, University of Illinois at Chicago College of Medicine, Chicago, Illinois, USA.; ^2^ Department of Emergency Medicine, University of Illinois at Chicago College of Medicine, Chicago, Illinois, USA.; ^3^ Department of Medicine, University of Illinois at Chicago College of Medicine, Chicago, Illinois, USA.; ^4^ Department of Medical Education, University of Illinois at Chicago College of Medicine, Chicago, Illinois, USA.; ^5^ Department of Sociology, College of Liberal Arts and Sciences, University of Illinois at Chicago, Chicago, Illinois, USA.; ^6^ Department of Obstetrics & Gynecology, University of Illinois at Chicago College of Medicine, Chicago, Chicago, Illinois, USA.

**Keywords:** academic medicine faculty, COVID-19 pandemic, work–life balance, health sciences, gender

## Abstract

**Background:**

Work and home stress, productivity, and self-care of academic medicine faculty in Spring 2021 was contrasted to faculty's experience in the Spring of 2020, both of which were relatively compared with the prepandemic period.

**Methods:**

A 93-question survey was sent to academic medicine faculty at an urban public university medical center in March 2020 and again in March 2021. Demographic, family, and academic characteristics, work distribution and productivity before and during the pandemic, perceived stress related to work and home activities, and self-care data compared with the prepandemic period were collected. Differences were assessed using chi-square or Fisher exact tests. Student *t*-test was used for the difference in mean values, while logistic regression was used to determine predictors of work stress.

**Results:**

Two hundred thirty-one faculty completed the survey in Spring 2020 and 118 faculty responded in Spring 2021. The proportion of faculty reporting increased work and home stress decreased in Spring 2021 compared with Spring 2020. A higher proportion of women compared with men reported increased work stress in both surveys. In Spring 2021, work stress decreased significantly for men but not for women. Home stress decreased significantly for women in Spring 2021 but remained stable for the men faculty. Research productivity increased for both genders in Spring 2021, but a greater percentage of women reported disturbed sleep and diet. There were no differences in home stress levels between genders when caring for young children.

**Conclusions:**

Men faculty are more likely to adapt to the “new normal” by lowering work stressors and increasing productivity, whereas women's continued high work stress and increased productivity may occur at the expense of decreased self-care. The challenges associated with having young children continue to affect the productivity and well-being of all faculty.

## Introduction

Work–life balance has been one of the primary concerns for faculty, particularly women faculty, for decades and the COVID-19 pandemic has upended any balance previously achieved.^1^ Several studies have documented the loss in scholarly research productivity especially by women faculty in academia during the pandemic.^2–4^ We previously demonstrated that during the first 6 months of the pandemic, the impact on academic faculty was extremely heterogeneous. Early- and mid-career individuals were impacted negatively by increased workloads, stress, and decreased self-care, while tenured professors in more senior roles and likely men were less affected.^5^

Similar studies have also focused on the pandemic's first several months and found a disproportional effect on women faculty.^6–9^ The Spring of 2020 was marked by government-issued stay-at-home orders, abrupt transitions to remote learning in an environment fraught with uncertainties, inadequate personal protection, and an overloaded health care system. The loss of childcare, closure of schools, insufficient support from administrations, and increased service-related activities directly affected productivity.^7^ Academic medicine faculty faced the additional stress of remote teaching and advising while working in a health care environment with unprecedented personal risk and increased emotional burdens while taking pay cuts.^9^ In this population, up to 20% of physicians considered leaving the workforce or reduced work hours, while more than 50% of faculty reported perceived decreased productivity and worried that the pandemic impacted their career development.^6^

A year later, we lived and worked in a hybrid world of remote and in-person activities, a better understanding of the COVID-19 virus, and a vaccine. The persistence of both the pandemic and numerous pandemic mitigation strategies has shaped a new environment in which we live and work. Literature has focused on the initial impact of the pandemic but not on how the continued presence of COVID-19 has affected faculty. Therefore, as we continue to navigate this environment, we felt it was important to evaluate how this unique population of academic medicine faculty has adapted to this “new normal.”

Our goal for this study was to compare the responses of academic medicine faculty regarding work stress, home stress, and self-care strategies from the beginning of the pandemic and 1 year later, as well as to capture gender differences in the Spring of 2021 and evaluate the impact of home activities on work stress.

We hypothesized that by Spring of 2021, faculty stress levels, regardless of gender, would have decreased in both work- and home-related activities, while academic productivity increased. We further suspected that a greater percentage of women compared with men, especially women with young children, would continue to report increased work and home stress compared with the prepandemic period and that home stress directly affected work stress and productivity.

## Methods

### Study design and survey instrument

A cross-sectional survey was conducted among faculty at the University of Illinois at Chicago (UIC), which is a large urban public university that comprised 16 academic schools and colleges. The survey was initially sent out *via* e-mail listservs between September and November 2020 and asked respondents about stress, productivity, and self-care at the beginning of the pandemic (Spring 2020). The survey (Supplementary Data) and results of the responses from this first period of the pandemic have been previously reported.^5^

The same survey was subsequently sent to the entire UIC faculty in March 2021 asking respondents about their current stress and productivity (Spring 2021) compared with the prepandemic time period. All faculty, regardless of if they had previously responded to the Spring 2020 survey, were invited to participate. To ensure anonymity, affiliation data were collected at the college level and any demographic group with fewer than five responses was merged into a larger category. The study was deemed exempt by the UIC Office for the Protection of Research Subjects (Protocol No. 2020-0853).

### Survey participants and instrument

Only respondents who were faculty in the College of Medicine (COM) were included in this analysis for two reasons. In our previous work,^5^ almost 50% of respondents were from the COM, comprising the largest group from the health sciences at UIC. Second, clinicians, especially physicians, faced additional burdens during the pandemic including the risk of COVID-19 from direct patient care, loss of clinical revenue from the closing of clinics/operating rooms, and loss of productivity due to halts on research activities. Those who did not indicate rank (*n* = 2) and those who completed less than 50% of the survey were excluded from analyses (*n* = 2). Sex was used as a proxy if respondents did not indicate their gender (*n* = 2).

The survey included the following domains and the number of questions in each topic:

- Demographics: 17
- Home Life Characteristics: 9
- Childcare/Aging Adult: 7
- Work–Life Characteristics: 16
- Administrative Activities: 3
- Clinical Productivity: 16
- Research Activities and Productivity: 22
- Teaching Activities and Productivity: 10
- Work Stress: 9
- Home Stress: 5
- Self-Care: 5
- Organization Culture: 9

Responses to stress-related questions were recorded on an 8-item and 5-item Likert scale and summed into composite work and home stress scores, respectively as previously described.^5^ Individuals with composite stress scores of 0–1 were considered “low stress.” Those with composite scores of ≥2 (*i.e*., two or more responsibilities reported as “more stressful”) were considered “high stress” for work- or home-related stress characteristics, respectively.

### Data analyses

The distributions of personal, family, and professional characteristics, and work and home stressors are presented as frequencies or bar charts and compared by chi-square tests or Fisher exact tests by gender (women vs. men) and by time period (Spring 2021 vs. Spring 2020). Age was presented as median [interquartile range] and analyzed by the Mann–Whitney rank-sum test.

Since the data set for both Spring 2020 and 2021 were not necessarily from the same individual over time, we combined data from Spring 2020 and 2021 into a pooled sample. We performed a series of multivariable logistic regression models to assess predictors of increased cumulative work or home stress as well as the various work and home characteristics. We used time period (Spring 2021 vs. Spring 2020) as a predictor to determine differences in stress level by time. Gender, career stage, faculty with children ≤12 years old, and time (Spring 2021 vs. Spring 2020) were included as predictors. Career stage was categorized as early or late career stage.

Based on our previous work,^5^ assistant professors, associate professors, lecturers, and instructors were classified as early-stage career faculty, while professors and adjunct/visiting professors were classified as late-stage career faculty. Age was excluded as a covariate in the regression model due to multicollinearity between age and faculty with or without children ≤12 years old. Interactions between predictor variables were also evaluated.

Research productivity (articles planned or submitted) between Spring 2020 and 2021 was determined using the Student *t*-test. Despite multiple comparisons (by gender and time period), we considered *p*-value <0.05 as statistically significant since our work was exploratory. Data were analyzed using SAS 9.4 software (Cary, NC).

## Results

### Respondent characteristics

A total of 231 respondents from the COM completed the survey in Spring of 2020, while 118 respondents completed the survey in Spring of 2021 (Table 1). Respondents in Spring 2021 were older than respondents in Spring 2020 (median age 54.0 vs. 47.0 years, *p* = 0.004). Within genders, age was the only demographic characteristics that showed significant difference between Spring 2020 and 2021. Men respondents in Spring 2021 were older than respondents in Spring 2020 (median age 63.0 vs. 53.0 years, *p* = 0.015). No differences were observed between time points and within genders for race/ethnicity, marital status, having a spouse/partner as frontline/essential worker, having children ≤12 years old, caring for or managing an aged/ill family member, current academic rank, tenure status, full-time status, or degree type.

**Table 1.** Personal, Family, and Professional Characteristics Stratified by Gender for Spring 2020 and Spring 2021 College of Medicine Respondents

Characteristic	Total			Women			Men		
	*SP20, N = 231*	*SP21, N = 118*	*p*	*SP20, n = 141*	*SP21, n = 60*	*p*	*SP20, n = 90*	*SP21, n = 58*	*p^a^*
Age, years, median [IQR]^b^	47 [39, 60]	54 [41, 65]	**0.004**	43.5 [37, 53.5]	46 [39, 55]	0.218	53 [44, 64]	63 [48, 68]	**0.015**
Race/ethnicity^c,d^			0.628			0.975			0.342
Black	12 (5.2)	3 (2.5)		9 (6.4)	3 (5.1)		3 (3.3)	0 (0.0)	
White	169 (73.5)	91 (77.8)		103 (73.6)	43 (72.9)		66 (73.4)	48 (82.8)	
Asian	36 (15.7)	18 (15.4)		19 (13.6)	9 (15.2)		17 (18.9)	9 (15.5)	
Other^e^	13 (5.6)	5 (4.3)		9 (6.4)	4 (6.8)		4 (4.4)	1 (1.7)	
Marital status^c,d^			0.135			0.091			0.818
Single	26 (11.4)	7 (5.9)		19 (13.7)	4 (6.6)		7 (7.9)	3 (5.2)	
Married or cohabitating	180 (79.0)	94 (79.7)		104 (74.8)	43 (71.7)		76 (85.4)	51 (87.9)	
Divorced/separated/widowed	22 (9.6)	17 (14.4)		16 (11.5)	13 (21.7)		6 (6.7)	4 (6.9)	
Spouse/partner is a frontline or essential worker^c,d^			0.495			0.688			0.514
Yes	55 (30.6)	25 (26.6)		30 (28.8)	11 (25.6)		25 (32.9)	14 (27.4)	
No	125 (69.4)	69 (73.4)		74 (71.2)	32 (74.4)		51 (67.1)	37 (72.6)	
Children ≤12 years			0.488			0.676			0.196
Yes	81 (35.1)	37 (31.4)		52 (36.9)	24 (40.0)		29 (32.2)	13 (22.4)	
No	150 (64.9)	81 (68.6)		89 (63.1)	36 (60.0)		61 (67.8)	45 (77.6)	
Currently caring for or managing care for an aging and/or ill parent, spouse/partner?^c,d^			0.637			0.469			0.780
Yes	36 (15.6)	16 (13.7)		25 (17.7)	8 (13.6)		11 (12.2)	8 (13.8)	
No	195 (84.4)	101 (86.3)		116 (82.3)	51 (86.4)		79 (87.8)	50 (86.2)	
Current rank^c^			0.112			0.289			0.649
Lecturer/instructor	5 (2.2)	1 (0.9)		5 (3.5)	1 (1.7)		0 (0.0)	0 (0.0)	
Assistant professor	98 (42.4)	43 (36.4)		72 (51.1)	28 (46.6)		26 (28.9)	15 (25.9)	
Associate professor	46 (19.9)	31 (26.3)		30 (21.3)	18 (30.0)		16 (17.8)	13 (22.4)	
Professor	64 (27.7)	40 (33.9)		23 (16.3)	12 (20.0)		41 (45.5)	28 (48.3)	
Adjunct/visiting	18 (7.8)	3 (2.5)		11 (7.8)	1 (1.7)		7 (7.8)	2 (3.4)	
Tenured status^c,d^			0.482			0.121			0.428
Tenured	68 (29.6)	39 (33.3)		33 (23.6)	14 (23.3)		35 (38.9)	25 (43.9)	
On tenure track, not tenured	25 (10.9)	16 (13.7)		17 (12.1)	14 (23.3)		8 (8.9)	2 (3.5)	
Not on tenure track	137 (59.6)	62 (53.0)		90 (64.3)	32 (53.3)		47 (52.2)	30 (52.6)	
Full-time status^c,d^			0.366			0.987			0.320
Yes	196 (84.8)	94 (81.0)		122 (86.5)	51 (86.4)		74 (82.2)	43 (75.4)	
No	35 (15.2)	22 (19.0)		19 (13.5)	8 (13.6)		16 (17.8)	14 (24.6)	
Degree(s)^c,d^			0.765			0.922			0.728
Clinical degree^f^	94 (40.8)	43 (37.1)		55 (39.3)	22 (37.3)		39 (43.3)	21 (36.8)	
Nonclinical degree only^g^	101 (43.9)	53 (45.7)		62 (44.3)	26 (44.1)		39 (43.3)	27 (47.4)	
Dual degree^h^	35 (15.2)	20 (17.2)		23 (16.4)	11 (18.6)		12 (13.4)	9 (15.8)	

^a^ *p*-Values bolded are significant at <0.05.

^b^ Data shown as median [IQR] and analyzed by Mann–Whitney rank-sum test.

^c^ Data shown as *n* (%) and analyzed by chi-square test, or Fisher exact test if cell is <5.

^d^ Totals do not add up due to missing values.

^e^ Includes Native American or Alaska Native, Native Hawaiian or other Pacific Islander.

^f^ Clinical degree: DDS, DMD, MD, DO, PharmD.

^g^ Nonclinical degree: PhD, DrPH, EdD, or any master's degree or other degree.

^h^ Dual degree: Clinical degree plus a PhD or masters.

IQR, interquartile range; SP20, Spring 2020; SP21, Spring 2021.

Compared with the total population of COM faculty, for both years, our sample showed an overrepresentation of women and a lower representation of nonwhite faculty and tenured faculty among the responders than in the COM faculty body as a whole (Supplementary Table S1).

### Stress evolution from Spring 2020 to Spring 2021

**FIG. 1.**
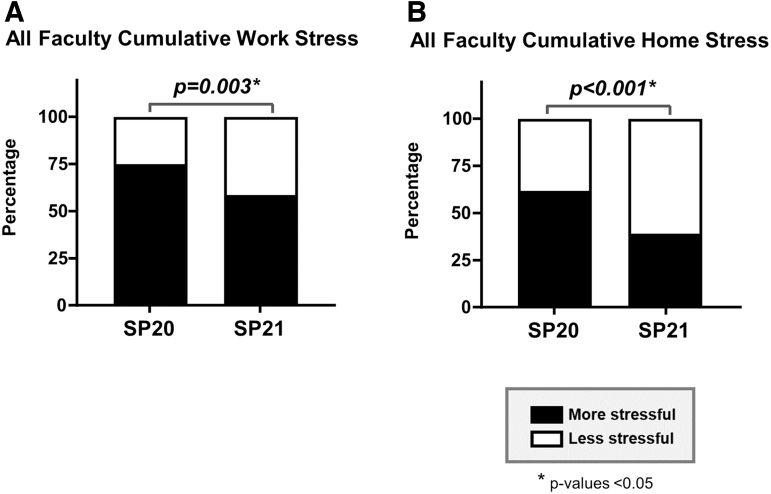
Proportion of faculty who reported feeling more stressed compared with the prepandemic period. The closed portion of bar graphs represents the proportion of faculty who reported feeling more stressed compared with the prepandemic period on the survey administered in SP20 compared with the survey administered in SP21. The open portion of the bars represents the proportion of faculty who reported no change or less stress. (**A**) All faculty cumulative work stress. (**B**) All faculty cumulative home stress. SP20, Spring 2020; SP21, Spring 2021.

Overall, a greater percentage of faculty reported decreased stress at work (42.2% vs. 26.0%, *p* = 0.003) (Fig. 1A) and home (62.7% vs. 40.4%, *p* < 0.001) (Fig. 1B) in Spring 2021 compared with Spring 2020.

### Work stress and gender

**FIG. 2.**
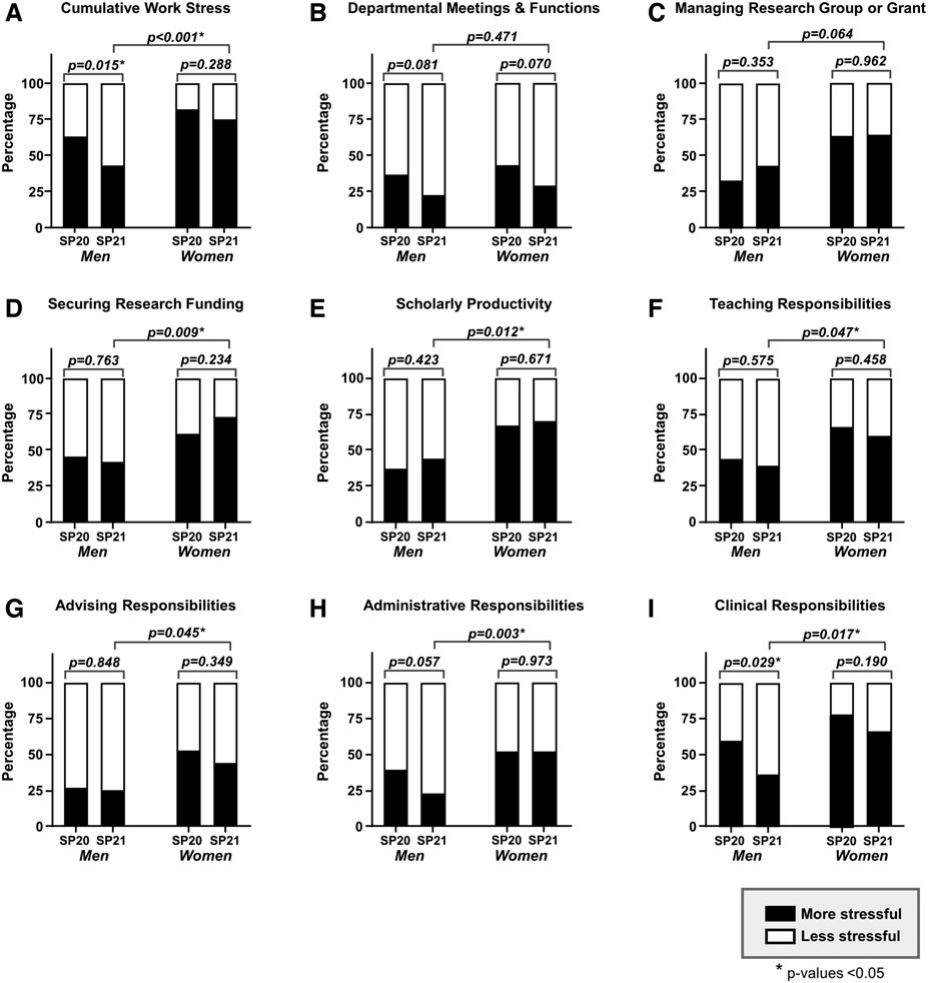
Proportion of men and women faculty who reported feeling more stressed by different work-related activities compared with the prepandemic period. The closed portion of bar graphs represents the proportion of faculty who reported feeling more stressed compared with the prepandemic period on the survey administered in SP20 compared with the survey administered in SP21. The open portion of the bars represents the proportion of faculty who reported no change or less stress. (**A**) Cumulative work stress. (**B**) Departmental meetings and functions. (**C**) Managing a research group or grant. (**D**) Securing research funding. (**E**) Scholarly productivity. (**F**) Teaching responsibilities. (**G**) Advising responsibilities. (**H**) Administrative responsibilities. (**I**) Clinical responsibilities.

Comparing within genders, a similar percentage of women reported increased work stress in Spring 2021 compared with Spring 2020 (74.1% vs. 81.0%, *p* = 0.288), while fewer men reported increased work stress when compared over the same time periods (41.8% vs. 62.8%, *p* = 0.015) (Fig. 2A). When evaluating specific components of work stress (Fig. 2B–I), no differences were seen within genders between the two time periods except for clinical responsibilities. Fewer men reported stress from clinical responsibilities in Spring 2021 compared with Spring 2020 (35.3% vs. 59.3%, *p* = 0.029), while for women, there was no difference between the time periods (65.5% vs. 77.6%, *p* = 0.190) (Fig. 2I). Specifically, for Spring of 2021, between gender comparison showed significantly that more women reported increased work stress compared with men (74.1% vs. 41.8%, *p* < 0.001) (Fig. 2A).

In addition, more women compared with men reported increased stress related to securing funding for research (72.2% vs. 41.2%, *p* = 0.009) (Fig. 2D), scholarly productivity (69.6% vs. 43.5%, *p* = 0.012) (Fig. 2E), teaching (59.1% vs. 38.3%, *p* = 0.047) (Fig. 2F), advising (43.8% vs. 24.5%, *p* = 0.045) (Fig. 2G), administrative responsibilities (51.0% vs. 22.6%, *p* = 0.003) (Fig. 2H), and clinical responsibilities (65.5% vs. 35.3%, *p* = 0.017) (Fig. 2I). No differences were seen between genders for department meetings or managing a research group (Fig. 2B, C).

### Home stress and gender

**FIG. 3.**
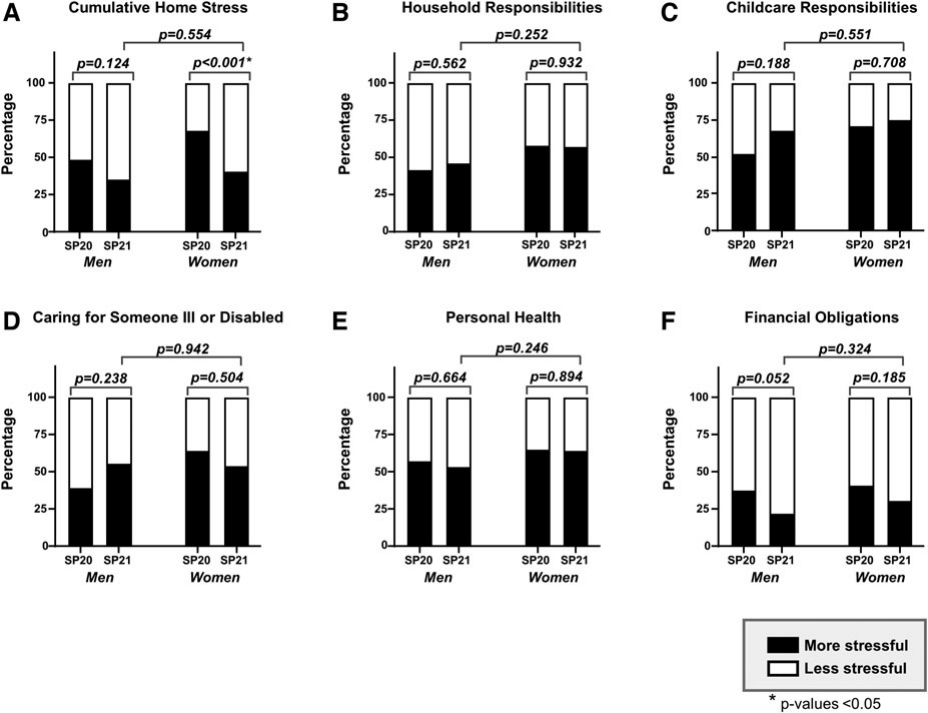
Proportion of men and women faculty who reported feeling more stressed by different home-related activities compared with the prepandemic period. The closed portion of bar graphs represents the proportion of faculty who reported feeling more stressed compared with the prepandemic period on the survey administered in SP20 compared with the survey administered in SP21. The open portion of the bars represents the proportion of faculty who reported no change or less stress. (**A**) Cumulative home stress. (**B**) Household responsibilities. (**C**) Childcare responsibilities. (**D**) Caring for someone ill or disabled. (**E**) Personal health. (**F**) Financial obligations.

Fewer women reported increased home stress in Spring 2021 compared with Spring 2020 (40.0% vs. 67.2%, *p* < 0.001), while for men, there was no significant difference (34.6% vs. 47.7%, *p* = 0.124) (Fig. 3A). When evaluating specific components of home stress (Fig. 3B–F), there were no differences both within genders and across time periods.

### Predictors of work stress

The significant predictors for cumulative work stress were time and gender (Table 2). Respondents in Spring 2021 were less likely to report cumulative work stress compared with Spring 2020 (odds ratio [OR] = 0.52, 95% confidence interval [CI]: 0.31–0.85), controlling for gender, career stage, and having a child ≤12 years old. Women were two times more likely to report cumulative work stress compared with men (OR = 2.45, 95% CI: 1.53–4.23), controlling for time, career stage, and having a child ≤12 years old.

**Table 2.** Predictors of Work-Related Stress for Spring 2020 and 2021 Pooled Years

Work stress characteristics	Spring 2020 and 2021, OR (95% CI)	*p*
Cumulative work stress^a^		
Time (SP21 vs. SP20)	**0.52 (0.31**–**0.85)**	**0.010**
Gender (women vs. men)	**2.45 (1.53**–**4.23)**	**0.001**
Career stage (early vs. late)^b^	1.73 (1.00–2.99)	0.050
Children ≤12 years (yes vs. no)	1.30 (0.74–2.32)	0.368
Departmental meetings and function		
Time (SP21 vs. SP20)	**0.50 (0.29**–**0.84)**	**0.010**
Gender (women vs. men)	1.07 (0.64–1.78)	0.803
Career stage (early vs. late)^b^	**1.97 (1.12**–**3.54)**	**0.021**
Children ≤12 (yes vs. no)	1.39 (0.83–2.33)	0.216
Managing research group or grant		
Time (SP21 vs. SP20)	1.17 (0.64–2.15)	0.614
Gender (women vs. men)	**2.74 (1.48**–**5.12)**	**0.001**
Career stage (early vs. late)^b^	1.73 (0.87–3.46)	0.117
Children ≤12 years (yes vs. no)	0.57 (0.28–1.13)	0.110
Securing research funding		
Time (SP21 vs. SP20)	1.22 (0.65–2.28)	0.540
Gender (women vs. men)	1.81 (0.98–3.38)	0.060
Career stage (early vs. late)^b^	**2.76 (1.40**–**5.53)**	**0.004**
Children ≤12 years (yes vs. no)	0.97 (0.48–1.93)	0.928
Scholarly productivity		
Time (SP21 vs. SP20)	1.30 (0.76–2.25)	0.351
Gender (women vs. men)	**2.64 (1.55**–**4.52)**	**0.001**
Career stage (early vs. late)^b^	**1.94 (1.09**–**3.49)**	**0.026**
Children ≤12 years (yes vs. no)	1.47 (0.82–2.62)	0.194
Teaching responsibilities		
Time (SP21 vs. SP20)	0.77 (0.45–1.30)	0.325
Gender (women vs. men)	**2.08 (1.24**–**3.48)**	**0.005**
Career stage (early vs. late)^b^	1.74 (1.00–3.06)	0.052
Children ≤12 (yes vs. no)	0.90 (0.52–1.54)	0.690
Advising responsibilities		
Time (SP21 vs. SP20)	0.77 (0.45–1.31)	0.337
Gender (women vs. men)	**2.43 (1.42**–**4.20)**	**0.001**
Career stage (early vs. late)^b^	**1.88 (1.04**–**3.45)**	**0.038**
Children ≤12 years (yes vs. no)	0.71 (0.40–1.24)	0.228
Administrative responsibilities		
Time (SP21 vs. SP20)	0.74 (0.44–1.22)	0.240
Gender (women vs. men)	**2.01 (1.22**–**3.32)**	**0.006**
Career stage (early vs. late)^b^	1.18 (0.69–2.03)	0.545
Children ≤12 years (yes vs. no)	**1.95 (1.16**–**3.30)**	**0.013**
Clinical responsibilities		
Time (SP21 vs. SP20)	**0.42 (0.22**–**0.80)**	**0.008**
Gender (women vs. men)	**2.14 (1.14**–**4.00)**	**0.017**
Career stage (early vs. late)^b^	**2.23 (1.11**–**4.49)**	**0.024**
Children ≤12 years (yes vs. no)	1.73 (0.89–3.44)	0.111

OR, CIs, and *p*-values bolded are significant at *p* < 0.05. There was no interaction between any of the predictors assessed.

^a^ Cumulative work stress: sum of the all the work stress characteristics.

^b^ Early-stage career includes assistant professors, associate professors, lecturers, and instructors; late-stage career includes professors and adjunct/visiting professors.

CI, confidence interval; OR, odds ratio.

For the breakdown of the various work stress characteristics, time and career stage were predictors of work stress due to departmental meetings and function. Respondents in Spring 2021 were less likely to report work stress due to departmental meetings and function compared with Spring 2020 (OR = 0.50, 95% CI: 0.29–0.84), controlling for gender, career stage, and having a child ≤12 years old, while early career stage faculty were almost two times more likely to report work stress due to departmental meetings and function (OR = 1.97, 95% CI: 1.12–3.54 vs. late career stage faculty), controlling for time, gender, and having a child ≤12 years old.

With regard to work stress due to managing a research group or grant, and teaching responsibilities, gender was the only predictor. Women were almost three times more likely to report work stress due to managing a research group or grant (OR = 2.74, 95% CI: 1.48–5.12) and two times more likely to report work stress due to teaching responsibilities (OR = 2.08, 95% CI: 1.24–3.48) compared with men, controlling for time, gender, and having a child ≤12 years old.

Career stage was the only predictor for work stress due to securing research funding. Early-stage career faculty were almost three times more likely to report work stress due to securing research funding (OR = 2.76, 95% CI: 1.40–5.53) compared with late career stage faculty, controlling for time, gender, and having a child ≤12 years old. Gender and career stage were predictors of work stress due to scholarly productivity and advising responsibilities.

Women were two times more likely to report work stress due to scholarly productivity (OR = 2.64, 95% CI: 1.55–4.52) and advising responsibilities (OR = 2.43, 95% CI: 1.42–4.20) compared with men, controlling for time, career stage, and having a child ≤12 years; while early-stage career faculty were almost two times more likely to report work stress due to scholarly productivity (OR = 1.94, 95% CI: 1.09–3.49) and advising responsibilities (OR = 1.88, 95% CI: 1.04–3.45) compared with late-stage career faculty, controlling for time, gender, and having a child ≤12 years old.

For administrative responsibilities, gender and having a child ≤12 years old were the only predictors, but not career stage. Compared with men, women were two times more likely to report work stress due to administrative responsibilities (OR = 2.01, 95% CI: 1.22–3.32), while faculty with children ≤12 years old were almost two times more likely to report stress due to administrative responsibilities (OR = 1.95, 95% CI: 1.16–3.30) compared with faculty without children ≤12 years old, holding other variables constant.

Time, gender, and career stage were predictors of work stress due to clinical responsibilities. Respondents in Spring 2021 were less likely (OR = 0.42, 95% CI: 0.22–0.80 vs. Spring 2020), while women (OR = 2.14, 95% CI: 1.14–4.00 vs. men) and early-stage career faculty (OR = 2.23, 95% CI: 1.11–4.49 vs. late-stage career) were two times more likely, to report work stress due to clinical responsibilities.

### Predictors of home stress

Time, career stage, and having a child ≤12 years old were predictors of cumulative home stress, but not gender (Table 3). Respondents in Spring 2021 were less likely to report cumulative home stress (OR = 0.38, 95% CI: 0.23–0.63) compared with Spring 2020, controlling for gender, career stage, and having a child ≤12 years. Early-stage career faculty (OR = 1.74, 95% CI: 1.02–2.98) vs. late-stage career faculty were almost two times more likely and faculty with children less than or equal to 12 years old (OR = 3.83, 95% CI: 2.24–6.69) vs. faculty without children less than or equal to 12 years old were almost four times more likely to report cumulative home stress while holding other variables constant.

**Table 3.** Predictors of Home-Related Stress for Spring 2020 and 2021 Pooled Years

Home stress characteristics	Spring 2020 and 2021, OR (95% CI)	*p*
Cumulative home stress^a^		
Time (SP21 vs. SP20)	**0.38 (0.23**–**0.63)**	**0.001**
Gender (women vs. men)	1.51 (0.92–2.49)	0.104
Career stage (early vs. late)^b^	**1.74 (1.02**–**2.98)**	**0.043**
Children ≤12 years (yes vs. no)	**3.83 (2.24**–**6.69)**	**0.001**
Managing household responsibilities		
Time (SP21 vs. SP20)	1.13 (0.68–1.89)	0.641
Gender (women vs. men)	1.46 (0.88–2.44)	0.142
Career stage (early vs. late)^b^	**1.80 (1.05**–**3.09)**	**0.032**
Children ≤12 years (yes vs. no)	**4.95 (2.89**–**8.69)**	**0.001**
Childcare		
Time (SP21 vs. SP20)	1.44 (0.63–3.40)	0.400
Gender (women vs. men)	1.32 (0.57–3.05)	0.512
Career-stage (early vs. late)^b^	1.76 (0.69–4.47)	0.233
Children ≤12 years (yes vs. no)	**9.34 (4.45**–**20.50)**	**0.001**
Currently caring for or managing care for an aging and/or ill parent, spouse, or partner		
Time (SP21 vs. SP20)	1.16 (0.50–2.71)	0.728
Gender (women vs. men)	1.48 (0.63–3.50)	0.374
Career-stage (early vs. late)^b^	2.27 (0.92–5.75)	0.079
Children ≤12 years (yes vs. no)	0.39 (0.14–1.02)	0.059
Personal health		
Time (SP21 vs. SP20)	0.89 (0.55–1.45)	0.644
Gender (women vs. men)	1.20 (0.74–1.93)	0.457
Career-stage (early vs. late)^b^	**1.98 (1.19**–**3.31)**	**0.009**
Children ≤12 years (yes vs. no)	1.16 (0.69–1.94)	0.582
Financial obligations		
Time (SP21 vs. SP20)	**0.52 (0.30**–**0.89)**	**0.018**
Gender (women vs. men)	0.92 (0.54–1.55)	0.744
Career-stage (early vs. late)^b^	**2.54 (1.41**–**4.70)**	**0.002**
Children ≤12 years (yes vs. no)	**2.65 (1.58**–**4.47)**	**0.001**

OR, CIs and *p*-values bolded are significant at *p* < 0.05. There was no interaction between any of the predictors assessed.

^a^ Cumulative home stress: sum of the all the home stress characteristics (managing household responsibilities, childcare, care of someone who is ill, personal health, financial obligations).

^b^ Early-stage career includes assistant professors, associate professors, lecturers, and instructors; late-stage career includes professors and adjunct/visiting professors.

Regarding specific home stress characteristics, career stage and having a child ≤12 years old predicted home stress due to managing household responsibilities. Early-stage career faculty were almost two times more likely to be stressed due to managing household responsibilities (OR = 1.80, 95% CI: 1.05–3.09), compared with late-stage career faculty. Faculty with children ≤12 years old were almost five times more likely to report home stress due to managing household responsibilities (OR = 4.95, 95% CI: 2.89–8.69) compared with faculty without children ≤12 years old, holding all other variables constant.

Having a child ≤12 years old was the only predictor of home stress related to childcare. Faculty with children ≤12 years old were nine times more likely to report stress due to childcare (OR = 9.34, 95% CI: 4.45–20.50) compared with faculty without children ≤12 years old, controlling for time, gender, and career stage.

Career stage was the only predictor of home stress due to personal health. Early-stage career faculty were nearly two times more likely to report stress due to personal health (OR = 1.98, 95% CI: 1.19–3.31) compared with late-stage career faculty, irrespective of time, gender, and having a child ≤12 years old.

However, time, career stage, and having a child ≤12 years old were predictors of home stress due to financial obligations. Faculty in Spring 2021 were less likely to report stress due to financial obligations (OR = 0.52, 95% CI: 0.30–0.89) compared with Spring 2020, while early-stage career faculty were two times more likely to report stress due to financial obligations (OR = 2.54, 95% CI: 1.41–4.70 vs. late-stage career faculty). Faculty with children ≤12 years old were almost three times more likely to report home stress due to financial obligations (OR = 2.65, 95% CI: 1.58–4.47 vs. faculty without children ≤12 years), controlling for time, gender, and career stage. No variable predicted home stress due to the care for or managing the care for an aged/ill family member.

In summary, having a child ≤12 years old predicted most of the home-related stress characteristics while gender predicted most work-related stress characteristics. There were no significant interactions between any of the predictive variables assessed (career stage, gender, children ≤12 years old) with time, between gender and career stage or children ≤12 years old, or between career stage and children ≤12 years old, for any of the work stress or home stress outcomes (data not shown).

### Research productivity

Research productivity based on the number of articles planned or submitted increased for all respondents in Spring 2021 compared with Spring 2020 and was observed within genders. Although men submitted more articles than women in Spring 2020 (1.68 ± 0.22 vs. 1.10 ± 0.18, *p* = 0.044), no differences were noted between genders by Spring 2021 (2.88 ± 0.58 vs. 2.52 ± 0.33, *p* = 0.563).

### Faculty self-care

No significant changes in overall self-care were reported with the exception that more faculty reported using mental health services in Spring 2021 compared with Spring 2020 (15.0% vs. 7.2%, *p* = 0.071). More men reported an increase in exercise in Spring 2021 than Spring 2020 (31.6% vs. 15.1%, *p* = 0.051), while more women used mental health services in Spring 2021 than in Spring 2020 (19.6% vs. 7.4%, *p* = 0.046). In Spring of 2021, a greater percentage of women respondents compared with men reported disturbed sleep (60.7% vs. 33.3%, *p* = 0.014) and disturbed diet (53.6% vs. 28.1%, *p* = 0.019).

## Discussion

In this survey assessing stress levels in subsequent years during the pandemic compared with prepandemic, academic medicine faculty reported decreased overall work and home stress in Spring 2021 compared with Spring 2020. Yet, for women, work stress did not change over this time, and they continued to experience more work stress than their male counterparts, while men reported decreased work stress in the same time frame. Both women and men reported increased research productivity and improved home stress, yet having a child aged 12 or younger in the home remained a consistent source of stress for both genders. Women's self-care such as sleep and diet has worsened, while men's self-care has marginally improved.

Our observed changes in stress are likely a reflection of both the alleviation of stressors and an adaption to the “new normal.” By 2021, the “new normal” of living with COVID-19 included knowledge of the virus, having methods to protect ourselves, as well as comfort in changing behaviors or activities to avoid COVID-19. Although men took on more home responsibility at the beginning of the pandemic,^10–12^ it was well documented that the bulk of household duties including day-to-day tasks, schooling, and childcare fell upon women.^12,13^ This distribution of workload was consistent even among physician parents.^14^ The decline in women reporting increased home stress as well as the stability in the number of men respondents supports the additional home and childcare responsibilities women took on. When services and resources were disrupted, in the case of the pandemic, the burden of this work fell upon women.^15^

As the disruptions have lessened, women's home stress has not necessarily gone away but simply declined compared with the extreme level of the prior year. In essence, our analysis provided a relative comparison of how men and women perceived their stress levels in Spring 2020 and in Spring 2021 compared with prepandemic, so within the lens of the “new normal,” there was a subtle diminishing of the home stress, but in the larger context, home stress levels were clearly higher than prepandemic especially for women.

We would have suspected a similar situation regarding work stress. However, we found there was greater stress. It was unexpected to find that almost 75% of surveyed women reported increased work stress and the odds of reporting increased work stress were four times greater for a woman. There are instances where early career continues to be a predictor of stress for departmental meetings, securing funding, and clinical responsibility. However, women continue to be a predictor of overall work stress, managing a research laboratory, scholarly productivity, teaching, advising, and administrative and clinical responsibilities. Specifically, for women, 6 of the 8 components of work stress characteristics had 50% or more reporting increased stress across both years, whereas for men, none of the components of work stress had 50% or more increase in stress.

This significant gender difference is noteworthy. Women took on a greater percentage of service and administrative roles at the beginning of the pandemic and many of these roles may have continued.^5^ Women are also known to take on more of these duties in academic environments in general^16,17^ and the gender differences may reflect this baseline difference or the age of respondents. As with our previous work,^5^ men who were more likely to be tenured professors, already had lower rates of work stress in service, administrative, and clinical duties. The persistent work stress in women could be also explained by cumulative stress, through which repeated exposure to the same or multiple stressors overtime can overwhelm individuals' ability to adapt.^18^

Yet it is under this stress that faculty have increased their productivity, although variable by gender. Women were less productive at article production at the beginning of the pandemic compared with men.^5^ We speculate that as home life was acutely disrupted, academic work, which had the least accountability, was sacrificed. Our work supports this as in 2020, several contributors to home stress such as childcare and managing household duties were predictors of increased work stress. Sharp et al.^19^ also found that women physicians with children were significantly more likely to report decreased academic productivity than men with children. Frank et al.^14^ noted that women physicians were more likely to work from home and voluntarily reduce their work hours during the pandemic.

The increase in productivity we observed in 2021 may reflect “catch up,” through which women are making up for lost productivity. In our analysis, those faculty on the tenure track but not tenured had higher levels of stress, suggesting that there is a pressure to achieve milestones within a now compressed time frame. Although many institutions have provided tenure rollback options, there is differential use of the rollback option by men and women, thereby further widening the gender promotion and pay disparities within academia^14^ and could potentially explain the persistent work stress seen among our women respondents.

We also believe the changes in stress were not simply the removal of stressors but the response and perception to stress. Humans are resilient by having the ability to adjust to changes and disturbances within systems.^20^ In other words, we adapt and compensate in different ways to increase and maintain productivity. While the way we compensate might be lifesaving in the short term, in the long term it may be harmful or unsustainable. In our survey, more women than men reported disturbances in sleep and diet as they entered Spring 2021. Our study suggests that women sacrifice self-care to manage stress and maintain productivity. In a survey of physicians, women were more likely to report greater family-to-work (family interfered with work roles) conflict than male peers and had higher rates of depressive and anxiety symptoms as well as fewer hours of sleep.^14^

Interestingly, childcare stress did not improve during the pandemic as we expected it would once policies for in-person learning were in place, vaccines were approved, and stay-at-home orders were lifted. The persistence of childcare stress may be because in-person learning had been erratic as COVID-19 cases disrupted classrooms or may also suggest that managing childcare has always been an issue and the pandemic just made it clearer. Even without full remote learning, it is stressful deciding what to do when quarantines happen, a child is ill, or other reasons for the loss of childcare options. It also may be a reflection that a vaccine was not approved for children younger than 12 years at the time of our survey.

Even more interesting is the role of faculty gender and childcare stress. In Spring of 2020, women faculty with younger children were more likely to report increased stress than men faculty with young children. Surprisingly, by Spring 2021, the faculty's gender did not matter. In other words, both men and women were impacted by the stress of having a young child at home who is dependent on others for care. Nevertheless, both men and women faculty reported more stress across both years relative to prepandemic due to caregiving responsibilities that further contributed to the disparate impact that certain faculty experienced. According to the National Academies of Sciences, Engineering, and Medicine (NASEM) survey on faculty, women managed 90% of the childcare demands early on in the pandemic.^21^

However, what our longitudinal analysis shows a year later is that childcare is not a gendered issue, but a caregiver issue and affects all faculty with young children.

With the increase in workload and blurring of boundaries, leading to decreased work effectiveness, some faculty were most disproportionately impacted. It is the faculty who intersect between high work stress and having a younger child at home who may be especially vulnerable. In our study, it is our women faculty, who are at risk. The issue of childcare during the pandemic impacted maternal labor force participation,^22,23^ through which women faculty physicians with children were more likely to reduce work hours or leave the workforce in the first 6 months of pandemic.^6^ Our results also show that the situation in 2020 was an acute crisis management, while a year later, individual-level compensatory mechanisms became more commonplace, including less personal care.

The problem is that with the social stressors, changing workloads and schedules, health risks, *etc*., the margin is very thin and there is no redundancy in compensatory mechanisms to allow one not to worry about another crisis coming along. Continued poor self-care in the context of high work stress to maintain productivity will contribute to burnout and failure to retain workers.^24–26^ Women in medicine are more likely to burnout due to work-related stress and often it is attributed to a broken health care system designed by and for men.^27^ The prepandemic work of Ecklund and Lincoln emphasizes this work–family conflict that disproportionately affects women, with academic scientists who are mothers paying the highest price.^28^ We will continue to risk women faculty pushed from academic medicine every time there is a direct challenge to this balance.

However, this issue may not be unique to just women. In their work, Ecklund and Lincoln present the case that academic science is not good for both women and men to have children.^28^ The concept of an “ideal scientist … a man, with a supportive wife who takes care of all his personal matters” whose work takes priority over family is not a realistic construct. Home life can be just as stressful for male faculty as our results show. Leaders can choose to acknowledge that home life directly affects work life and provide support for those faculty members to promote and retain a diverse faculty. However, Ecklund and Lincoln argue that institutions need structural change to accommodate this need to acknowledge the impact on home life.^28^

There are some limitations that should put the results in context. First, our findings are based on the small number of respondents from a single COM and so may not be representative of the greater population. It is also possible that those who responded in Spring of 2021 were not representative of the population surveyed in 2020. The 2021 respondents may represent a form of survivorship bias, through which those faculty who were extremely stressed or faced additional burdens may have left the workforce and could not be surveyed. We were unable to obtain data regarding faculty who took leave or left the university. In addition, the smaller number of total respondents in 2021 may represent nonresponse bias, by which those who have less stress may not have responded. Second, in the Spring of 2021, the sample of men was older than those in Spring of 2020.

In our previous work,^5^ older male professors experienced the pandemic differently in terms of less work and home stress. And finally, our survey examines the proportion of respondents on each survey and not individual trends over time with the same individuals. Responses were provided anonymously; thus, individual faculty responses between the two time periods could not be associated. We do believe though that there is a significant overlap between the individuals who completed the survey in Spring of 2020 and Spring of 2021.

Even with these limitations, the results are still valuable, especially when we examine the number of women respondents whose stressors have not changed. Our data here demonstrate that a year into the pandemic, academic medicine faculty are still dealing with many of the challenges that the NASEM highlighted in their report examining the impact COVID-19 had in 2020.^21^ Just as the individual has adapted to this pandemic, it is time for higher education to as well. As Emanuel et al.^29^ wrote in their viewpoint, “A National Strategy for the New Normal’ of Life With COVID,” COVID-19 is here to stay, and we need to create policies that focus on living with this virus. We echo a call for academic health centers to incorporate best practices from other academic settings to support faculty productivity.

Ellinas et al.^8^ articulated that “productivity and work-life balance predict each other … emphasizing the inseparable connections between work and life success.” We believe that even small steps taken to acknowledge the underrecognized burden of caregiving can minimize stress for junior faculty and allow for greater workplace productivity. During this postpandemic recovery, it is critical that academic institutions are proactive about a gender-responsive promotion and tenure plan. In addition, as faculty who had left the workforce may consider reentry, policies and procedures to accommodate their return would be critical. Just as the National Institutes of Health (NIH) have passed a policy to ensure that sex as a biological variable has been incorporated into the analysis, perhaps academic institutions need a policy to track gender benchmarking. These types of interventions will help to support women's careers not only during the extremes of a pandemic but in all cases as well.

## Conclusion

In our study, we highlight the impact of the pandemic on faculty home and work stress from Spring 2020 to Spring 2021. Overall, more faculty are reporting decreased stress in year 2 of the pandemic for both work and home environments, with improved productivity. However, almost 75% of surveyed women continue to have increased work stress and this percentage is significantly greater than men. Having young children at home continues to be a stressor for both women and men faculty. Women with high work stress and young children have a disproportionate burden and may be especially vulnerable. A year into this pandemic, these boundaries between work and home remain blurred, causing persistent challenges for women. With data already showing compromise to women's self-care, one needs to ask how much longer this is sustainable and where is the breaking point for women faculty to leave academic medicine.

Overall, the societal and institutional inequities exposed by the pandemic may have lasting negative consequences on women faculty. It is imperative that institutions acknowledge that one's home stressors, especially childcare, risk the productivity and health of a gender-diverse workforce.

## Supplementary Material

Supplemental data

Supplemental data

## Abbreviations Used

CI: confidence interval
COM: College of Medicine
IQR: interquartile range
NASEM: National Academies of Sciences, Engineering, and Medicine
NIH: National Institutes of Health
OR: odds ratio
SP20: Spring 2020
SP21: Spring 2021
UIC: University of Illinois at Chicago
