# Supplementary material for: Work–Life Balance and Academic Productivity Among College of Medicine Faculty During the Evolution of the COVID-19 Pandemic: The New Normal
Source: Womens Health Rep (New Rochelle). 2023 Jul 18;4(1):367–80. doi: 10.1089/whr.2023.0007 (PMC10354727; doi:10.1089/whr.2023.0007)
Supplement: Supplemental data [file Suppl_Data.pdf]

Supplementary Table S1

Worklife Balance Productivity And Coping Strategies - Spring 2021

The following survey may seem familiar to some faculty (West campus faculty in particular). Please continue and retake the survey.

Thank you for your time!

Demographics	
1) What is your age in years?	__________________________________
2) What is your sex?	◯ Female<br>◯ Male<br>◯ Intersex<br>◯ Other
2b) Please specify sex	__________________________________
3) What is your gender/gender identity?	◯ Woman<br>◯ Man<br>◯ Transgender Woman / Trans Feminine<br>◯ Transgender Man / Trans Masculine<br>◯ Non-Binary / Genderqueer / Gender Fluid<br>◯ Prefer to self-describe<br>◯ Prefer not to say
3b) Please specify gender/gender identity	__________________________________
4) Are you of Hispanic, Latino, or of Spanish origin?	◯ Yes<br>◯ No
5) How would you describe yourself?	◯ American Indian or Alaska Native<br>◯ Asian<br>◯ Black or African American<br>◯ Native Hawaiian or Other Pacific Islander<br>◯ White<br>◯ Other
5b) How would you describe yourself?	__________________________________
6) What is your marital status?	◯ Single (never married)<br>◯ Married, or Cohabitating<br>◯ Widowed<br>◯ Divorced<br>◯ Separated

7) What is your current rank?	◯ Lecturer<br>◯ Instructor<br>◯ Assistant Professor<br>◯ Associate Professor<br>◯ Professor<br>◯ Adjunct<br>◯ Visiting<br>◯ Other
7b) What is your rank?	__________________________________
8) You are currently:	◯ Tenured<br>◯ On tenure track, not tenured<br>◯ Not on tenure track
9) Are you full time?	◯ Yes<br>◯ No
9b) If part time please indicate % time.	__________________________________
10) What degree(s) do you hold? (Check all that apply)	☐ DDS or DMD<br>☐ MD<br>☐ PhD<br>☐ DrPH<br>☐ Masters<br>☐ PharmD<br>☐ Other
11) Your primary appointment is in the following school/college:	◯ Applied Health Sciences<br>◯ Dentistry<br>◯ Medicine<br>◯ Nursing<br>◯ Pharmacy<br>◯ Public Health<br>◯ Social Work<br>◯ Architecture, Design, and the Arts<br>◯ Business Administration<br>◯ Education<br>◯ Engineering<br>◯ Liberal Arts and Sciences<br>◯ Urban Planning and Public Affairs<br>◯ Council on Teacher Education<br>◯ Graduate College<br>◯ Honors College<br>◯ Other
10b) What is your degree?	__________________________________
11b) In what school/college is your primary appointment?	__________________________________
Home/family related	
Is your spouse/partner a frontline or essential worker?	◯ Yes<br>◯ No

Is your spouse/partner currently working remotely (from home) or has other flexible work arrangements in place?	◯ They always work remotely<br>◯ They are working remotely all of the time during the COVID-19 outbreak<br>◯ They are working remotely some of the time during the COVID-19 outbreak<br>◯ Their work cannot be done remotely<br>◯ They are currently not working
Is your spouse/partner's work affected in another way by the social distancing requirement due to COVID-19? (Check all that apply)	☐ No change<br>☐ Furloughed<br>☐ Temporarily laid off<br>☐ Position terminated<br>☐ Increased hours<br>☐ Decreased hours<br>☐ Lost pay<br>☐ Added pay<br>☐ Other
How is your spouse/partners' work affected?	__________________________________
How many children do you have in total?	____________________________
What are the age ranges of your children? (Check all that apply)	☐ 0-4 yrs<br>☐ 5-12 yrs<br>☐ 13-17 yrs<br>☐ 18-23 yrs<br>☐ 24 or older
Are you currently caring for or managing care for an aging and/or ill parent, spouse/partner, or other relative?	◯ Yes<br>◯ No
1) BEFORE the social distancing requirement to prevent the spread of COVID-19, who in your household did most of the housework such as cooking and cleaning?	◯ I did all of it<br>◯ I did most of it<br>◯ My spouse/partner and I shared the work equally<br>◯ My spouse/partner did most of it<br>◯ My spouse/partner did all of it<br>◯ Someone else did it
2) DURING the social distancing requirement to prevent the spread of COVID-19, who in your household currently does most of the housework such as cooking and cleaning?	◯ I do all of it<br>◯ I do most of it<br>◯ My spouse/partner and I share the work equally<br>◯ My spouse/partner does most of it<br>◯ My spouse/partner does all of it<br>◯ Someone else does it
3) Are there aspects of your home life that have impacted you positively as a result of government-required social distancing?	◯ Yes<br>◯ No<br>◯ Don't know
3b) If yes, please explain.	__________________________________

4) Are there aspects of your home life that have impacted you negatively as a result of government-required social distancing?

- ◯ Yes
- ◯ No
- ◯ Don't know

4b) If yes, please explain.

____

5) What services, if any, did you utilize to maintain a work-life balance PRIOR to COVID-19 were discontinued or reduced as a result of government-required social distancing? (Check all that apply)

- ☐ Nanny/babysitter
- ☐ Housecleaning services
- ☐ Lawncare services
- ☐ Cooking/meal-prep services
- ☐ Personal trainer
- ☐ Pet sitter
- ☐ Dog walker
- ☐ I did not use any of these services
- ☐ Other

5b) What other services? (Please specify)

____

Childcare

Childcare

1) Who in your household provides most of the childcare since the government began requiring social distancing to prevent the spread of COVID-19?

- ◯ I do all of it
- ◯ I do most of it
- ◯ My spouse/partner and I share the work equally
- ◯ My spouse/partner does most of it
- ◯ My spouse/partner does all of it
- ◯ Someone else does it
- ◯ All my children are old enough to be fairly independent

2) Compared to before the government began requiring social distancing to prevent the spread of COVID-19, how much time do you currently spend on childcare?

- ◯ More time compared to before COVID-19
- ◯ The same amount of time
- ◯ Less time compared to before COVID-19

3) Since schools have closed, who in your household is spending more time homeschooling your child(ren) or helping them with distance learning?

- ◯ I do most of it
- ◯ My spouse/partner and I share the work equally
- ◯ My spouse/partner does most of it
- ◯ My spouse/partner does all of it
- ◯ Someone else does it

4) To what extent do you feel pressure to do parent-led education activities with your child(ren)?

- ◯ A lot of pressure
- ◯ Some pressure
- ◯ Not very much pressure
- ◯ No pressure at all
- ◯ Don't Know / No Opinion

Work Characteristics

Scope of work
As you think about how you spend your time during an academic year, what percent of your typical work week did you spend on each of the following work-related activities PRIOR to the social distancing requirement due to COVID-19?

(The sum of these responses should be equal to your % FTE, see total at end of these questions. You may leave blank if not applicable)

Department meetings and functions	________________
Managing a research group or grant	________________
Managing a research laboratory	________________
Grant development and submission	________________
Scholarly productivity (e.g., writing, manuscripts,...)	________________
Teaching responsibilities	________________
Advising responsibilities	________________
Committee and/ or administrative responsibilities	________________
Clinical responsibilities	________________
Other responsibilities- percentage of time spent? (Please specify)	________________
Please describe your other responsibilities.	____________________
Total for Scope of work (Before COVID-19)<br><br>Total should match your % FTE.	________________
Remote work	
1) PRIOR to the social distancing requirement to prevent the spread of COVID-19, what percent of your work was done remotely?<br><br>Note: please submit an integer	________________
2) PRIOR to the social distancing requirement to prevent the spread of COVID-19, what were your Department or Division official policies or accepted norms regarding remote work? (Check all that apply)	☐ No remote work during business hours<br>☐ Administrative time may be conducted from home<br>☐ Work can be conducted anywhere as long as productivity is maintained<br>☐ No formal policy<br>☐ Other
2b) Other remote work policy (Please specify)	________________

Please indicate your agreement or disagreement with the following statements:

	Strongly disagree	Disagree	Neither agree nor disagree	Agree	Strongly agree
My department/unit is a place where individual faculty may comfortably raise personal and/or family responsibilities when scheduling departmental/unit obligations.	◯	◯	◯	◯	◯
I feel that the climate and opportunities for female faculty in my department/unit are at least as good as those for male faculty.	◯	◯	◯	◯	◯
I feel that the climate and opportunities for faculty of color in my department/unit are at least as good as those for white faculty.	◯	◯	◯	◯	◯
Since the government has required social distancing to prevent the spread of COVID-19, my department/unit has been supportive of working parents.	◯	◯	◯	◯	◯
My department/unit expects me to work more compared to before COVID-19.	◯	◯	◯	◯	◯
UIC has a strong commitment to diversity, equity, and inclusion.	◯	◯	◯	◯	◯
I have opportunities at UIC for professional success that are similar to those of my colleagues.	◯	◯	◯	◯	◯
UIC provides sufficient programs and resources to foster the success of a diverse faculty.	◯	◯	◯	◯	◯
I have to work harder than others to be valued equally at UIC.	◯	◯	◯	◯	◯

Since the government began requiring social distancing to prevent the spread of COVID-19, how has your productivity changed for each aspect of your work?

	Significantly increased	Increased	No change	Decreased	Significantly decreased	NA
Departmental meetings and functions	◯	◯	◯	◯	◯	◯
Managing a research group or grant	◯	◯	◯	◯	◯	◯
Managing a research laboratory	◯	◯	◯	◯	◯	◯
Grant development and submission	◯	◯	◯	◯	◯	◯
Scholarly productivity (e.g., writing, manuscripts,...)	◯	◯	◯	◯	◯	◯
Teaching responsibilities	◯	◯	◯	◯	◯	◯
Advising responsibilities	◯	◯	◯	◯	◯	◯
Committee and/ or administrative responsibilities	◯	◯	◯	◯	◯	◯
Clinical responsibilities	◯	◯	◯	◯	◯	◯

Are there other aspects of work that have changed since Covid-19? If so, please describe.

____

Clinical

1) Please indicate what your clinical work involves. (Check all that apply)

- ☐ Outpatient
- ☐ Hospital based
- ☐ Procedural
- ☐ Non- procedural
- ☐ Laboratory
- ☐ Other

1b) What other clinical work are you involved in?

____

2) Does your clinical work involve direct patient care?

- ◯ Yes
- ◯ No

3) Does your clinical work involve face-to-face care of COVID-19+ patients or Persons Under Investigation (PUIs)?

- ◯ Yes
- ◯ No
- ◯ Other

3b) Please specify your clinical work involving COVID + patients or PUIs.

____

4) During the social distancing requirement to prevent the spread of COVID-19, were there changes made by your department leaders that promoted your clinical productivity?

- ◯ Yes
- ◯ No

4b) If yes, please explain.	__________________________________
5) During the social distancing requirement to prevent the spread of COVID-19, were there changes made by your department leaders that decreased your clinical productivity?	◯ Yes<br>◯ No
5b) If yes, please explain.	__________________________________
6) Were there aspects of telehealth that you found positive?	◯ Yes<br>◯ No<br>◯ Unsure
7) During the social distancing requirement to prevent the spread of COVID-19, did your clinical work involve telehealth medicine?	◯ Yes<br>◯ No<br>◯ N/A
7b) If yes, please explain.	__________________________________
8) Were there aspects of telehealth that you found negative?	◯ Yes<br>◯ No<br>◯ Unsure
8b) If yes, please explain.	__________________________________
9) Would you want to incorporate telehealth in your practice once social distancing is no longer required?	◯ Yes<br>◯ No<br>◯ Unsure
Research	
1) Research<br>What type of research work do you conduct? (Check all that apply)	☐ Basic sciences<br>☐ Clinical<br>☐ Informatics<br>☐ Health services<br>☐ Humanities<br>☐ Arts<br>☐ Other
1b) What other types of research?	____________________________
2) In what ways, if any, has your research work been affected since the government began requiring social distancing to prevent the spread of COVID-19? Please describe.	____________________________
3) How many grants were you planning to submit in the calendar year 2020?	____________________________

4) How many abstracts for conferences were you planning to submit in the calendar year 2020?

____

5) How many manuscripts were you planning to submit in the calendar year 2020?

____

6) Are you planning to submit any COVID-19 related grants?

◯ Yes
◯ No

7) Are you planning to submit any COVID-19 related manuscripts?

◯ Yes
◯ No

8) How many grants for conferences have you submitted in the calendar year 2020?

____

9) How many abstracts for conferences have you submitted in the calendar year 2020?

____

10) How many manuscripts have you submitted in the calendar year 2020?

____

11) How many COVID-19 related grants have you submitted?

____

12) How many COVID-19 related manuscripts have you submitted?

____

13) Since the government began requiring social distancing to prevent the spread of COVID-19, have there been aspects of remote work that have increased your research productivity?

◯ Yes
◯ No

13b) If yes, please explain.

____

14) Since the government began requiring social distancing to prevent the spread of COVID-19, have there been aspects of remote work that have decreased your research productivity?

◯ Yes
◯ No

14b) If yes, please explain.

____

15) Are there aspects of remote work related to your research you would want to continue once social distancing is no longer required?

◯ Yes
◯ No
◯ Unsure

15b) If yes, please describe.

____

Teaching

1) Were there aspects of remote teaching that you found positive?	◯ Yes<br>◯ No<br>◯ Unsure
1b) If yes, please explain.	__________________________________
2) Were there aspects of remote teaching that you found negative?	◯ Yes<br>◯ No<br>◯ Unsure
2b) If yes, please explain.	__________________________________
3) Would you want to continue remote teaching once social distancing is no longer required?	◯ Yes<br>◯ No<br>◯ Unsure<br>◯ N/A
4) In what ways, if any, did teaching remotely because of the social distancing requirement to prevent the spread of COVID-19 impact your work? Please describe.	__________________________________
5) Did/Do you need to convert your class or teaching sessions to remote teaching because of the social distancing requirement to prevent the spread of COVID-19?	◯ Yes<br>◯ No<br>◯ N/A<br>◯ Other
5b) What other way were you affected by remote teaching due to COVID -19?	__________________________________
5c) Please indicate the time it took you to convert your class or teaching sessions to remote teaching. Note: Please submit the number of hours.	____________________________

Please indicate your current level of stress, if any, related to each aspect of your work compared to before the social distancing requirement to prevent the spread of COVID-19. (Choices: Much more stressful, More stressful, Neutral, Less stressful, Much less stressful, N/A)

	Much more stressful	More stressful	Neutral	Less stressful	Much less stressful	N/A
Departmental meetings and functions	◯	◯	◯	◯	◯	◯
Managing a research group or grant (e.g., finances, personnel)	◯	◯	◯	◯	◯	◯
Securing funding for research	◯	◯	◯	◯	◯	◯

Scholarly productivity	○	○	○	○	○	○
Teaching responsibilities	○	○	○	○	○	○
Advising responsibilities	○	○	○	○	○	○
Committee and/or administrative responsibilities	○	○	○	○	○	○
Clinical responsibilities	○	○	○	○	○	○

Are there other aspects of work that have changed your stress level? If so, please describe.

____

Please indicate your current level of stress, if any, related to each aspect of your home life compared to before the social distancing requirement to prevent the spread of COVID-19. (Choices: Much more stressful, More stressful, Neutral, Less stressful, Much less stressful, N/A)

	Much more stressful	More stressful	Neutral	Less stressful	Much less stressful	N/A
Managing household responsibilities	○	○	○	○	○	○
Childcare	○	○	○	○	○	○
Care of someone who is ill, disabled, aging, and/or in need of special services	○	○	○	○	○	○
Your health	○	○	○	○	○	○
Financial obligations	○	○	○	○	○	○

Self-care

For each of the following, how have the following changed for you during the social distancing requirement to prevent the spread of COVID-19?

1) Sleep
○ Disturbed
○ No Change
○ Improved

2) Diet
○ Disturbed
○ No Change
○ Improved

3) Exercise
○ Increased
○ No Change
○ Decreased

4) Use of mental health services
○ Increased
○ No Change
○ Decreased

5) Emotional support from friends, colleagues, family
○ Increased
○ No Change
○ Decreased

General Comments

Please provide any additional comments here.

____

Thank you for completing this survey.

Work-Life Balance, Productivity and Coping Strategies during the COVID Pandemic

Please complete the survey below.

Thank you!

Demographics

1) What is your age in years? __________________________________

2) What is your sex?
- ◯ Female
- ◯ Male
- ◯ Preferred response not listed

3) What is your gender/gender identity?
- ◯ Cis Woman
- ◯ Cis Man
- ◯ Trans Woman
- ◯ Trans Man
- ◯ Gender Fluid/Gender Nonconforming
- ◯ Non-Binary/Genderqueer
- ◯ Preferred response not listed

2b) Please specify sex __________________________________

3b) Please specify gender __________________________________

4) Are you of Hispanic, Latino, or of Spanish origin?
- ◯ Yes
- ◯ No

5) How would you describe yourself?
- ◯ American Indian or Alaska Native
- ◯ Asian
- ◯ Black or African American
- ◯ Native Hawaiian or Other Pacific Islander
- ◯ White
- ◯ Other

5b) How would you describe yourself? __________________________________

6) What is your marital status?
- ◯ Single (never married)
- ◯ Married, or Cohabitating
- ◯ Widowed
- ◯ Divorced
- ◯ Separated

7) What is your current rank?
- ◯ Lecturer
- ◯ Instructor
- ◯ Assistant Professor
- ◯ Associate Professor
- ◯ Professor
- ◯ Adjunct
- ◯ Visiting
- ◯ Other

7b) What is your rank?	__________________________
8) You are currently:	◯ Tenured<br>◯ Not tenured and on tenure track<br>◯ Not on tenure track
9) Are you full time?	◯ Yes<br>◯ No
9b) If part time please indicate % time.	__________________________
10) What degree(s) do you hold? (Check all that apply)	☐ DDS or DMD<br>☐ MD<br>☐ PhD<br>☐ DrPH<br>☐ Masters<br>☐ PharmD<br>☐ Other
10b) What is your degree?	__________________________
11) Your primary appointment is in the following school/college:	◯ Applied Health Sciences<br>◯ Dentistry<br>◯ Medicine<br>◯ Nursing<br>◯ Pharmacy<br>◯ Public Health<br>◯ Social Work<br>◯ Other
11b) In what school/college is your primary appointment?	__________________________
Home/family related	
Is your spouse/partner a frontline or essential worker?	◯ Yes<br>◯ No
Is your spouse/partner currently working remotely (from home) or has other flexible work arrangements in place?	◯ They always work remotely<br>◯ They are working remotely all of the time during the COVID-19 outbreak<br>◯ They are working remotely some of the time during the COVID-19 outbreak<br>◯ Their work cannot be done remotely<br>◯ They are currently not working
Is your spouse/partner's work affected in another way by the social distancing requirement due to COVID-19? (Check all that apply)	☐ No change<br>☐ Furloughed<br>☐ Temporarily laid off<br>☐ Position terminated<br>☐ Increased hours<br>☐ Decreased hours<br>☐ Lost pay<br>☐ Added pay<br>☐ Other

How is your spouse/partners' work affected?	__________________________________
How many children do you have in total?	____________________________
What are the age ranges of your children? (Check all that apply)	☐ 0-4 yrs<br>☐ 5-12 yrs<br>☐ 13-17 yrs<br>☐ 18-23 yrs<br>☐ 24 or older
Are you currently caring for or managing care for an aging and/or ill parent, spouse/partner, or other relative?	○ Yes<br>○ No
1) BEFORE the social distancing requirement to prevent the spread of COVID-19, who in your household did most of the housework such as cooking and cleaning?	○ I did all of it<br>○ I did most of it<br>○ My spouse/partner and I shared the work equally<br>○ My spouse/partner did most of it<br>○ My spouse/partner did all of it<br>○ Someone else did it
2) DURING the social distancing requirement to prevent the spread of COVID-19, who in your household currently does most of the housework such as cooking and cleaning?	○ I do all of it<br>○ I do most of it<br>○ My spouse/partner and I share the work equally<br>○ My spouse/partner does most of it<br>○ My spouse/partner does all of it<br>○ Someone else does it
3) Are there aspects of your home life that have impacted you positively as a result of government-required social distancing?	○ Yes<br>○ No<br>○ Don't know
3b) If yes, please explain.	__________________________________
4) Are there aspects of your home life that have impacted you negatively as a result of government-required social distancing?	○ Yes<br>○ No<br>○ Don't know
4b) If yes, please explain.	__________________________________
5) What services, if any, did you utilize to maintain a work-life balance PRIOR to COVID-19 were discontinued or reduced as a result of government-required social distancing? (check all that apply)	☐ Nanny/babysitter<br>☐ Housecleaning services<br>☐ Lawncare services<br>☐ Cooking/meal-prep services<br>☐ Personal trainer<br>☐ Pet sitter<br>☐ Dog walker<br>☐ I did not use any of these services<br>☐ Other

5b) What other services? (Please specify)

____

Childcare

Childcare

1) Who in your household provides most of the childcare since the government began requiring social distancing to prevent the spread of COVID-19?

- ◯ I do all of it
- ◯ I do most of it
- ◯ My spouse/partner and I share the work equally
- ◯ My spouse/partner does most of it
- ◯ My spouse/partner does all of it
- ◯ Someone else does it
- ◯ All my children are old enough to be fairly independent

2) Compared to before the government began requiring social distancing to prevent the spread of COVID-19, how much time do you currently spend on childcare?

- ◯ More time compared to before COVID-19
- ◯ The same amount of time
- ◯ Less time compared to before COVID-19

3) Since schools have closed, who in your household is spending more time homeschooling your child(ren) or helping them with distance learning?

- ◯ I do most of it
- ◯ My spouse/partner and I share the work equally
- ◯ My spouse/partner does most of it
- ◯ My spouse/partner does all of it
- ◯ Someone else does it

4) To what extent do you feel pressure to do parent-led education activities with your child(ren)?

- ◯ A lot of pressure
- ◯ Some pressure
- ◯ Not very much pressure
- ◯ No pressure at all
- ◯ Don't Know / No Opinion

Work Characteristics

Scope of work
As you think about how you spend your time during an academic year, what percent of your typical work week did you spend on each of the following work-related activities PRIOR to the social distancing requirement due to COVID-19?

(The sum of these responses should be equal to your % FTE, see total at end of these questions. You may leave blank if not applicable)

Department meetings and functions

____

Managing a research group or grant

____

Managing a research laboratory

____

Grant development and submission

____

Scholarly productivity (e.g., writing, manuscripts,...)

____

Teaching responsibilities

____

Advising responsibilities ______________________________

Committee and/ or administrative responsibilities ______________________________

Clinical responsibilities ______________________________

Other responsibilities- percentage of time spent? (Please specify) ______________________________

Please describe your other responsibilities. ______________________________

Total for Scope of work (Before COVID-19) ______________________________

Total should match your % FTE.

Remote work

1) PRIOR to the social distancing requirement to prevent the spread of COVID-19, what percent of your work was done remotely? ______________________________

Note: please submit an integer

2) PRIOR to the social distancing requirement to prevent the spread of COVID-19, what were your Department or Division official policies or accepted norms regarding remote work? (Check all that apply)

- ☐ No remote work during business hours
- ☐ Administrative time may be conducted from home
- ☐ Work can be conducted anywhere as long as productivity is maintained
- ☐ No formal policy
- ☐ Other

2b) Other remote work policy (Please specify) ______________________________

Please indicate your agreement or disagreement with the following statements:

	Strongly disagree	Disagree	Neither agree nor disagree	Agree	Strongly agree
My department/unit is a place where individual faculty may comfortably raise personal and/or family responsibilities when scheduling departmental/unit obligations.	○	○	○	○	○

I feel that the climate and opportunities for female faculty in my department/unit are at least as good as those for male faculty.	○	○	○	○	○
I feel that the climate and opportunities for faculty of color in my department/unit are at least as good as those for white faculty.	○	○	○	○	○
Since the government has required social distancing to prevent the spread of COVID-19, my department/unit has been supportive of working parents.	○	○	○	○	○
My department/unit expects me to work more compared to before COVID-19.	○	○	○	○	○
UIC has a strong commitment to diversity, equity, and inclusion.	○	○	○	○	○
I have opportunities at UIC for professional success that are similar to those of my colleagues.	○	○	○	○	○
UIC provides sufficient programs and resources to foster the success of a diverse faculty.	○	○	○	○	○
I have to work harder than others to be valued equally at UIC.	○	○	○	○	○

Since the government began requiring social distancing to prevent the spread of COVID-19, how has your productivity changed for each aspect of your work?

	Significantly increased	Increased	No change	Decreased	Significantly decreased	NA
Departmental meetings and functions	○	○	○	○	○	○
Managing a research group or grant	○	○	○	○	○	○
Managing a research laboratory	○	○	○	○	○	○
Grant development and submission	○	○	○	○	○	○
Scholarly productivity (e.g., writing, manuscripts,...)	○	○	○	○	○	○

Teaching responsibilities	◯	◯	◯	◯	◯	◯
Advising responsibilities	◯	◯	◯	◯	◯	◯
Committee and/ or administrative responsibilities	◯	◯	◯	◯	◯	◯
Clinical responsibilities	◯	◯	◯	◯	◯	◯

Are there other aspects of work that have changed since Covid-19? If so, please describe.

____

Clinical

1) Please indicate what your clinical work involves. (Check all that apply)

☐ Outpatient
☐ Hospital based
☐ Procedural
☐ Non- procedural
☐ Laboratory
☐ Other

1b) What other clinical work are you involved in?

____

2) Does your clinical work involve direct patient care?

◯ Yes
◯ No

3) Does your clinical work involve face-to-face care of COVID-19+ patients or Persons Under Investigation (PUIs)?

◯ Yes
◯ No
◯ Other

3b) Please specify your clinical work involving COVID + patients or PUIs.

____

4) During the social distancing requirement to prevent the spread of COVID-19, were there changes made by your department leaders that promoted your clinical productivity?

◯ Yes
◯ No

4b) If yes, please explain.

____

5) During the social distancing requirement to prevent the spread of COVID-19, were there changes made by your department leaders that decreased your clinical productivity?

◯ Yes
◯ No

5b) If yes, please explain.

____

6) Were there aspects of telehealth that you found positive?

◯ Yes
◯ No
◯ Unsure

7) During the social distancing requirement to prevent the spread of COVID-19, did your clinical work involve telehealth medicine?	◯ Yes<br>◯ No<br>◯ N/A
7b) If yes, please explain.	__________________________________
8) Were there aspects of telehealth that you found negative?	◯ Yes<br>◯ No<br>◯ Unsure
8b) If yes, please explain.	__________________________________
9) Would you want to incorporate telehealth in your practice once social distancing is no longer required?	◯ Yes<br>◯ No<br>◯ Unsure
Research	
1) Research<br>What type of research work do you conduct? (Check all that apply)	☐ Basic sciences<br>☐ Clinical<br>☐ Informatics<br>☐ Health services<br>☐ Other
1b) What other types of research?	____________________________
2) In what ways, if any, has your research work been affected since the government began requiring social distancing to prevent the spread of COVID-19? Please describe.	____________________________
3) How many grants were you planning to submit during March - June 2020?	____________________________
4) How many abstracts for conferences were you planning to submit during March - June 2020?	____________________________
5) How many manuscripts were you planning to submit during March - June 2020?	____________________________
6) Are you planning to submit any COVID-19 related grants?	◯ Yes<br>◯ No
7) Are you planning to submit any COVID-19 related manuscripts?	◯ Yes<br>◯ No
8) How many abstracts for conferences have you submitted during March - June 2020?	____________________________
9) How many manuscripts have you submitted during March - June 2020?	____________________________

10) How many COVID-19 related grants have you submitted?	__________________________________
11) How many COVID-19 related manuscripts have you submitted?	__________________________________
12) Since the government began requiring social distancing to prevent the spread of COVID-19, have there been aspects of remote work that have increased your research productivity?	◯ Yes<br>◯ No
12b) If yes, please explain.	____
13) Since the government began requiring social distancing to prevent the spread of COVID-19, have there been aspects of remote work that have decreased your research productivity?	◯ Yes<br>◯ No
13b) If yes, please explain.	____
14) Are there aspects of remote work related to your research you would want to continue once social distancing is no longer required?	◯ Yes<br>◯ No<br>◯ Unsure
14b) If yes, please describe.	____
Teaching	
1) Were there aspects of remote teaching that you found positive?	◯ Yes<br>◯ No<br>◯ Unsure
1b) If yes, please explain.	____
2) Were there aspects of remote teaching that you found negative?	◯ Yes<br>◯ No<br>◯ Unsure
2b) If yes, please explain.	____
3) Would you want to continue remote teaching once social distancing is no longer required?	◯ Yes<br>◯ No<br>◯ Unsure<br>◯ N/A

4) In what ways, if any, did teaching remotely because of the social distancing requirement to prevent the spread of COVID-19 impact your work? Please describe. ______________________________

5) Did/Do you need to convert your class or teaching sessions to remote teaching because of the social distancing requirement to prevent the spread of COVID-19?

- ◯ Yes
- ◯ No
- ◯ N/A
- ◯ Will start remote teaching in the fall
- ◯ Other

5b) What other way were you affected by remote teaching due to COVID -19? ______________________________

5c) Please indicate the time it took you to convert your class or teaching sessions to remote teaching. Note: Please submit the number of hours. ________________________

Please indicate your current level of stress, if any, related to each aspect of your work compared to before the social distancing requirement to prevent the spread of COVID-19. (Choices: Much more stressful, More stressful, Neutral, Less stressful, Much less stressful, N/A)

	Much more stressful	More stressful	Neutral	Less stressful	Much less stressful	N/A
Departmental meetings and functions	◯	◯	◯	◯	◯	◯
Managing a research group or grant (e.g., finances, personnel)	◯	◯	◯	◯	◯	◯
Securing funding for research	◯	◯	◯	◯	◯	◯
Scholarly productivity	◯	◯	◯	◯	◯	◯
Teaching responsibilities	◯	◯	◯	◯	◯	◯
Advising responsibilities	◯	◯	◯	◯	◯	◯
Committee and/or administrative responsibilities	◯	◯	◯	◯	◯	◯
Clinical responsibilities	◯	◯	◯	◯	◯	◯

Are there other aspects of work that have changed your stress level? If so, please describe. ______________________________

Please indicate your current level of stress, if any, related to each aspect of your work compared to before the social distancing requirement to prevent the spread of COVID-19. (Choices: Much more stressful, More stressful, Neutral, Less stressful, Much less stressful, N/A)

	Much more stressful	More stressful	Neutral	Less stressful	Much less stressful	N/A

Managing household responsibilities	◯	◯	◯	◯	◯	◯
Childcare	◯	◯	◯	◯	◯	◯
Care of someone who is ill, disabled, aging, and/or in need of special services	◯	◯	◯	◯	◯	◯
Your health	◯	◯	◯	◯	◯	◯
Financial obligations	◯	◯	◯	◯	◯	◯

Self-care

For each of the following, how have the following changed for you during the social distancing requirement to prevent the spread of COVID-19?

1) Sleep
◯ Disturbed
◯ No Change
◯ Improved

2) Diet
◯ Disturbed
◯ No Change
◯ Improved

3) Exercise
◯ Increased
◯ No Change
◯ Decreased

4) Use of mental health services
◯ Increased
◯ No Change
◯ Decreased

5) Emotional support from friends, colleagues, family
◯ Increased
◯ No Change
◯ Decreased

General Comments

Please provide any additional comments here.

____

Would you be willing to be interviewed for future studies related to work life?
◯ Yes
◯ No
◯ Unsure

Thank you for completing this survey.
