# Supplementary material for: Work–Life Balance and Academic Productivity Among College of Medicine Faculty During the Evolution of the COVID-19 Pandemic: The New Normal
Source: Womens Health Rep (New Rochelle). 2023 Jul 18;4(1):367–80. doi: 10.1089/whr.2023.0007 (PMC10354727; doi:10.1089/whr.2023.0007)
Supplement: Supplemental data [file Suppl_TableS1.pdf]

Supplement Table: Survey Responders Compared to Total College of Medicine Faculty by Year.

	2020		2021	
	Responding Faculty % (n)	Total Faculty % (n)	Responding Faculty % (n)	Total Faculty % (n)
**<u>Gender</u>**				
Men	39.1 (90)	60.6 (340)	50.4 (59)	60.2 (339)
Women	60.9 (140)	39.4 (221)	49.6 (58)	39.8 (224)
**<u>Race</u>**				
White	73.5 (169)	56.5 (317)	77.8 (91)	55.1 (310)
Black	5.2 (12)	3.2 (18)	2.6 (3)	3.2 (18)
Asian	15.7 (36)	25.1 (141)	15.4 (18)	26.3 (148)
Other	5.7 (13)	15.1 (85)	4.2 (5)	15.5 (87)
**<u>Tenure Status</u>**				
Non-Tenured	59.6 (137)	31.4 (176)	53.0 (62)	28.4 (160)
Tenure-Track, Non-tenure	10.9 (25)	25.1 (141)	13.7 (16)	27.9 (157)
Tenured	29.6 (68)	43.5 (244)	33.3 (39)	43.7 (246)
